# Supplementary material for: PDZD-8 and TEX-2 regulate endosomal PI(4,5)P2 homeostasis via lipid transport to promote embryogenesis in C. elegans
Source: Nat Commun. 2021 Oct 18;12:6065. doi: 10.1038/s41467-021-26177-z (PMC8523718; doi:10.1038/s41467-021-26177-z)
Supplement: Supplementary file 1 — Supplementary Information [file 41467_2021_26177_MOESM1_ESM.pdf]

Supplementary Information

PDZD-8 and TEX-2 regulate endosomal PI(4,5)P_2 homeostasis via lipid transport to promote embryogenesis in *C. elegans*

Darshini Jeyasimman[1], Bilge Ercan[1]*, Dennis Dharmawan[1]*, Tomoki Naito[1], Jingbo Sun[1], Yasunori Saheki[1,2]**

[1]Lee Kong Chian School of Medicine, Nanyang Technological University, 308232, Singapore

[2]Department of Molecular Physiology, Faculty of Life Sciences, Kumamoto University, Kumamoto 860-8556, Japan

*These authors contributed equally to this work

**Address correspondence to: yasunori.saheki@ntu.edu.sg (Y.S.)

- **Supplementary Figures 1-9**
- **Supplementary Table 1**

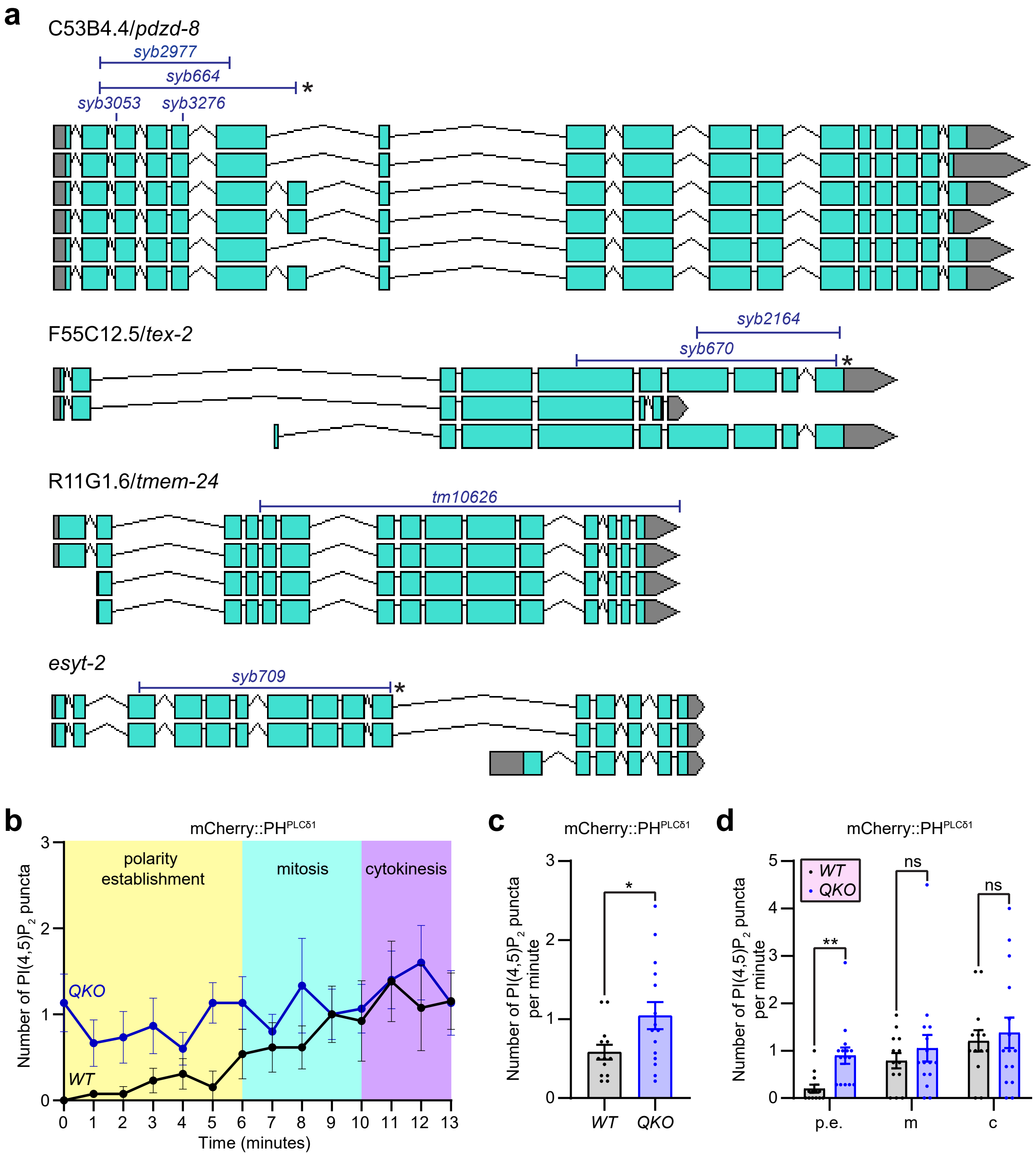

Supplementary Fig. 1 Ectopic accumulation of PI(4,5)P$_2$ in the absence of SMP proteins in *C. elegans* early embryos. a Schematics of *C. elegans* SMP protein genes, C53B4.4/*pdzd-8*, F55C12.5/*tex-2*, R11G1.6/*tmem-24*, and *esyt-2*. Deletions (*syb2977, syb664, syb2164, syb670, tm10626, syb709*) and mutations (*syb3053, syb3276*) are indicated by blue lines. * denote premature stop codons in respective deletion alleles. **b** Time-course analysis of the number of PI(4,5)P$_2$-positive puncta, as assessed by mCherry::PH^{PLCδ1}, during polarity establishment (0-6 minutes), mitosis (6-10 minutes), and cytokinesis (10-13 minutes) phases for wild-type control (*WT*) and *quadruple knock-out* [*QKO (pdzd-8; tex-2; tmem-24; esyt-2)*] early embryos as shown in Fig. 1b [mean ± SEM, n=13 embryos (*WT*), n=15 embryos (*QKO*)]. **c** Quantification of the number of PI(4,5)P$_2$-positive puncta, as assessed by mCherry::PH^{PLCδ1}, per minute during early embryogenesis. Comparisons between *WT* and *QKO* early embryos are shown [mean ± SEM, n=13 embryos (*WT*), n=15 embryos (*QKO*); two-tailed unpaired Student's t-test, *p=0.0330]. **d** Quantification of the number of PI(4,5)P$_2$-positive puncta, as assessed by mCherry::PH^{PLCδ1}, per minute in each phase of the early embryogenesis as indicated for *WT* and *QKO* embryos [mean ± SEM, n=13 embryos (*WT*), n=15 embryos (*QKO*); two-tailed unpaired Student's t-test, **p=0.001690 (p.e.), ns denotes not significant]. p.e., m and c denote polarity establishment, mitosis, and cytokinesis, respectively.

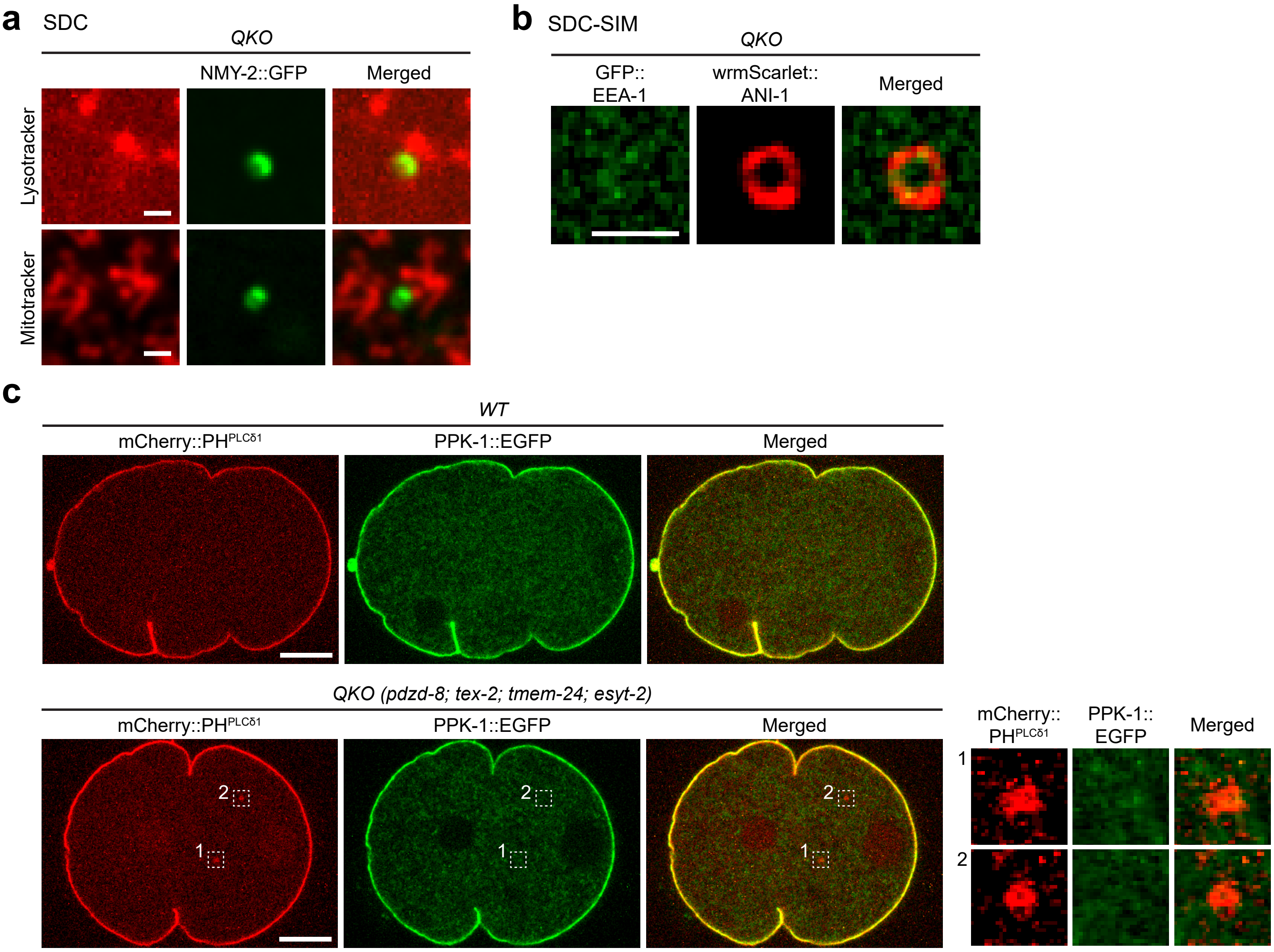

Supplementary Fig. 2 Aberrant recruitment of proteins involved in actomyosin contraction but not PIP5K in the absence of SMP proteins. a Live spinning disc confocal (SDC) images of early embryos from mutants lacking all the four SMP proteins [*quadruple knock-out: QKO (pdzd-8; tex-2; tmem-24; esyt-2)*] expressing NMY-2-tagged with GFP (NMY-2::GFP) that were stained by either lysotracker (lysosomes) or mitotracker (mitochondria). Note the absence of co-localization between NMY-2::GFP and these organelle markers. Scale bars, 1μm. **b** Live SDC images from a *QKO* early embryo co-expressing GFP-tagged EEA-1 [(GFP::EEA-1), early endosome marker] and wrmScarlet-tagged ANI-1 (wrmScarlet::ANI-1). Note the absence of co-localization between GFP::EEA-1 and wrmScarlet::ANI-1. Scale bar, 1μm. **c** Left: Representative live SDC images of equatorial planes of early embryos from wild-type control (*WT*) (top panel) and *QKO* mutants (bottom panel), co-expressing PI(4,5)P_2 biosensor (mCherry::$PH^{PLC\delta1}$) and EGFP-tagged PPK-1 (PPK-1::EGFP). Images of each row are from time-lapse movies of the same embryos at polarity establishment phase. Right: Magnified insets, showing PI(4,5)P_2-positive puncta, as determined by the presence of mCherry::$PH^{PLC\delta1}$, that are outlined by indicated white dotted boxes. Note the absence of PPK-1::EGFP on cytoplasmic PI(4,5)P_2 puncta. Scale bars, 10μm.

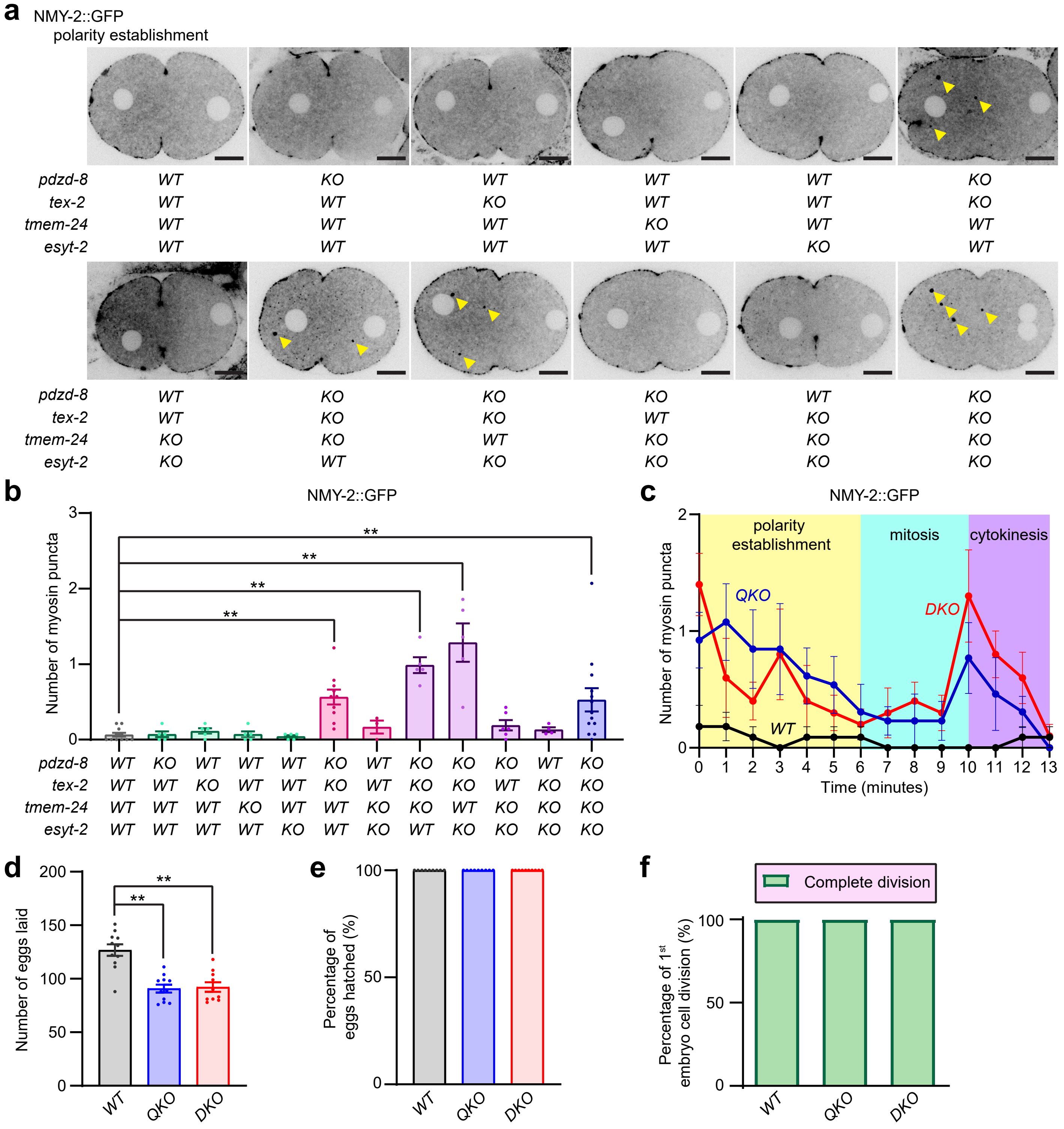

Supplementary Fig. 3 Systematic analysis of single, double, triple and quadruple knock-outs of SMP proteins reveals redundant function of PDZD-8 and TEX-2. a Representative live spinning disc confocal (SDC) images of equatorial planes of early embryos from wild-type control (*WT*) and indicated SMP protein mutants, expressing NMY-2-tagged with GFP (NMY-2::GFP). Images are from polarity establishment phase. Note the presence of ectopic NMY-2::GFP puncta, indicated by yellow arrowheads, in mutant embryos that are simultaneously depleted with PDZD-8 and TEX-2. Scale bars, 10μm. **b** Quantification of the number of NMY-2::GFP puncta during early embryogenesis. Comparisons between *WT* and indicated mutant embryos are shown [mean ± SEM, n=11 embryos (*WT*), n=5 embryos (*pdzd-8*), n=5 embryos (*tex-2*), n=5 embryos (*tmem-24*), n=5 embryos (*esyt-2*), n=10 embryos (*pdzd-8; tex-2*), n=3 embryos (*tmem-24; esyt-2*), n=5 embryos (*pdzd-8; tex-2; tmem-24*), n=5 embryos (*pdzd-8; tex-2; esyt-2*), n=6 embryos (*pdzd-8; tmem-24; esyt-2*), n=4 embryos (*tex-2; tmem-24; esyt-2*), n=13 embryos (*QKO*); Dunnett's multiple comparisons test, **p=0.0058 (*pdzd-8; tex-2*), **p<0.0001 (*pdzd-8; tex-2; tmem-24*), **p<0.0001 (*pdzd-8; tex-2; esyt-2*), **p=0.0066 (*QKO*)]. **c** Time-course analysis of the number of NMY-2::GFP puncta during polarity establishment (0-6 minutes), mitosis (6-10 minutes), and cytokinesis (10-13 minutes) phases for *WT*, *QKO* and *DKO* early embryos as shown in Fig. 3a [mean ± SEM, n=11 embryos (*WT*), n=13 embryos (*QKO*), n=10 embryos (*DKO*)]. **d** Quantification of the number of eggs laid by *WT* animals, *QKO* mutants and *DKO* mutants [mean ± SEM, n=11 animals (*WT*), n=11 animals (*QKO*), n=10 animals (*DKO*); Dunnett's multiple comparisons test, **p<0.0001 (*QKO*), **p<0.0001 (*DKO*)]. **e** Quantification of the percentage of eggs successfully hatched in indicated strains as in (d) [mean ± SEM, n=11 animals (*WT*), n=11 animals (*QKO*), n=10 animals (*QKO*)]. **f** Quantification of the first embryonic cell divisions in early embryos from indicated strains [n=11 embryos (*WT*), n=14 embryos (*QKO*), n=11 embryos (*DKO*)].

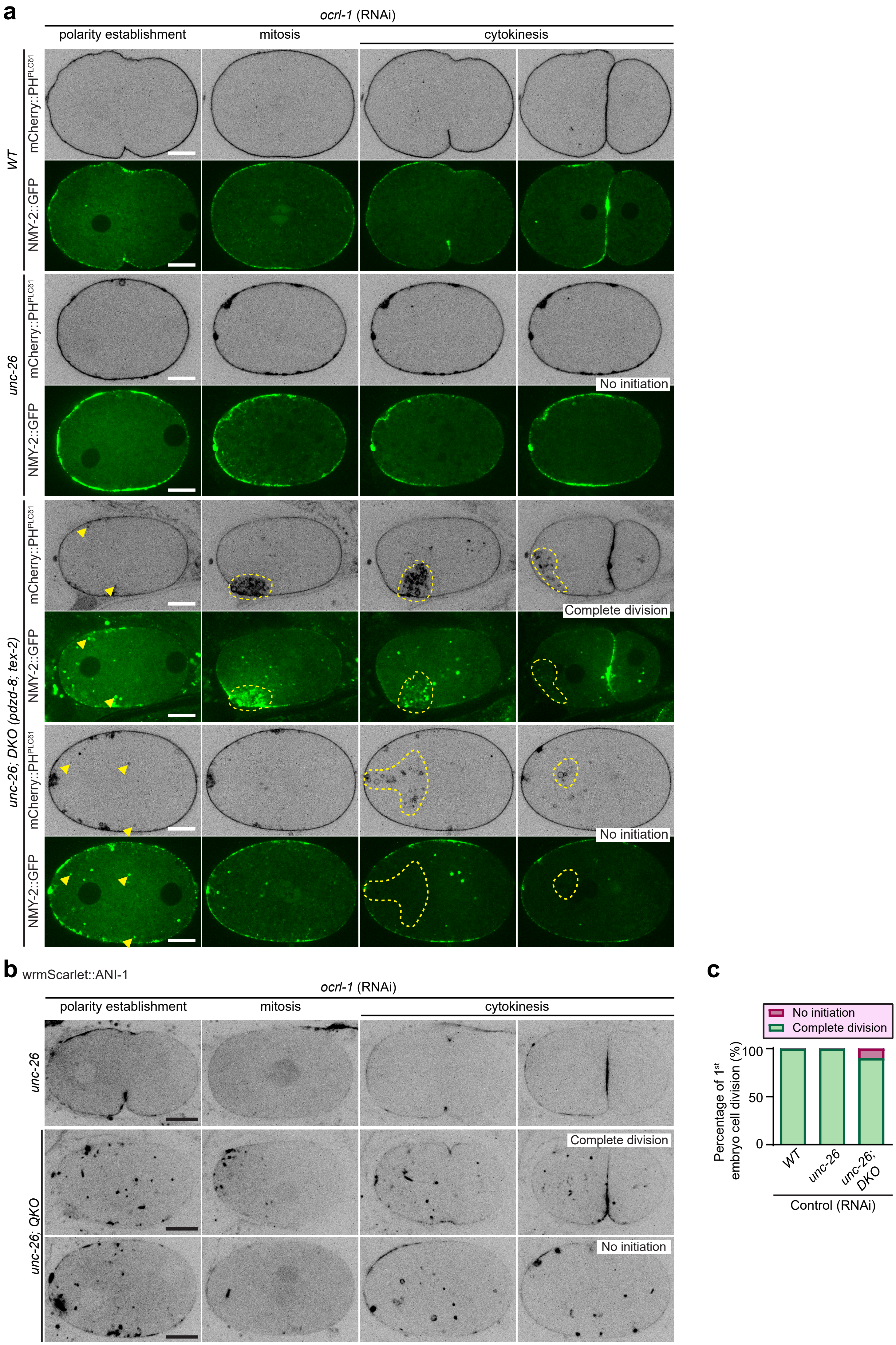
a
ocrl-1 (RNAi)
polarity establishment
mitosis
cytokinesis
WT
mCherry::PH^PLCδ1
NMY-2::GFP
unc-26
No initiation
unc-26; DKO (pdzd-8; tex-2)
Complete division
No initiation
b
wrmScarlet::ANI-1
ocrl-1 (RNAi)
polarity establishment
mitosis
cytokinesis
unc-26
unc-26; QKO
Complete division
No initiation
c
No initiation
Complete division
Percentage of 1st embryo cell division (%)
100
50
0
WT
unc-26
unc-26; DKO
Control (RNAi)

Supplementary Fig. 4 Simultaneous depletion PDZD-8, TEX-2, and PI(4,5)P_2 phosphatases results in massive accumulation of endosomal PI(4,5)P_2 and anillin. a Representative live spinning disc confocal (SDC) images of equatorial planes of early embryos from OCRL-1 RNAi-treated wild-type control (*WT*) animals, OCRL-1 RNAi-treated *unc-26* mutants and OCRL-1 RNAi-treated *unc-26; double knock-out* [*DKO (pdzd-8; tex-2)*] mutants, co-expressing PI(4,5)P_2 biosensor (mCherry::$PH^{PLC\delta1}$) and NMY-2-tagged with GFP (NMY-2::GFP). Images of each row are from time-lapse movies of the same embryos at different phases as indicated. Yellow arrowheads in polarity establishment phase indicate PI(4,5)P_2-positive vesicles that are co-localized with NMY-2::GFP. Yellow dotted lines in mitosis and cytokinesis phases indicate clustering of PI(4,5)P_2-positive vesicles that are only present in early embryos from OCRL-1 RNAi-treated *unc-26; DKO* mutants. Scale bars, 10μm. **b** Representative live SDC images of equatorial planes of early embryos from OCRL-1 RNAi-treated *unc-26* mutants and OCRL-1 RNAi-treated *unc-26; quadruple knock-out* [*QKO (pdzd-8; tex-2; tmem-24; esyt-2)*] mutants, expressing wrmScarlet-tagged ANI-1 (wrmScarlet::ANI-1). Images of each row are from time-lapse movies of the same embryos at different phases as indicated. Scale bars, 10μm. **c** Quantification of the first embryonic cell divisions in early embryos from indicated conditions [n=10 embryos for all conditions].

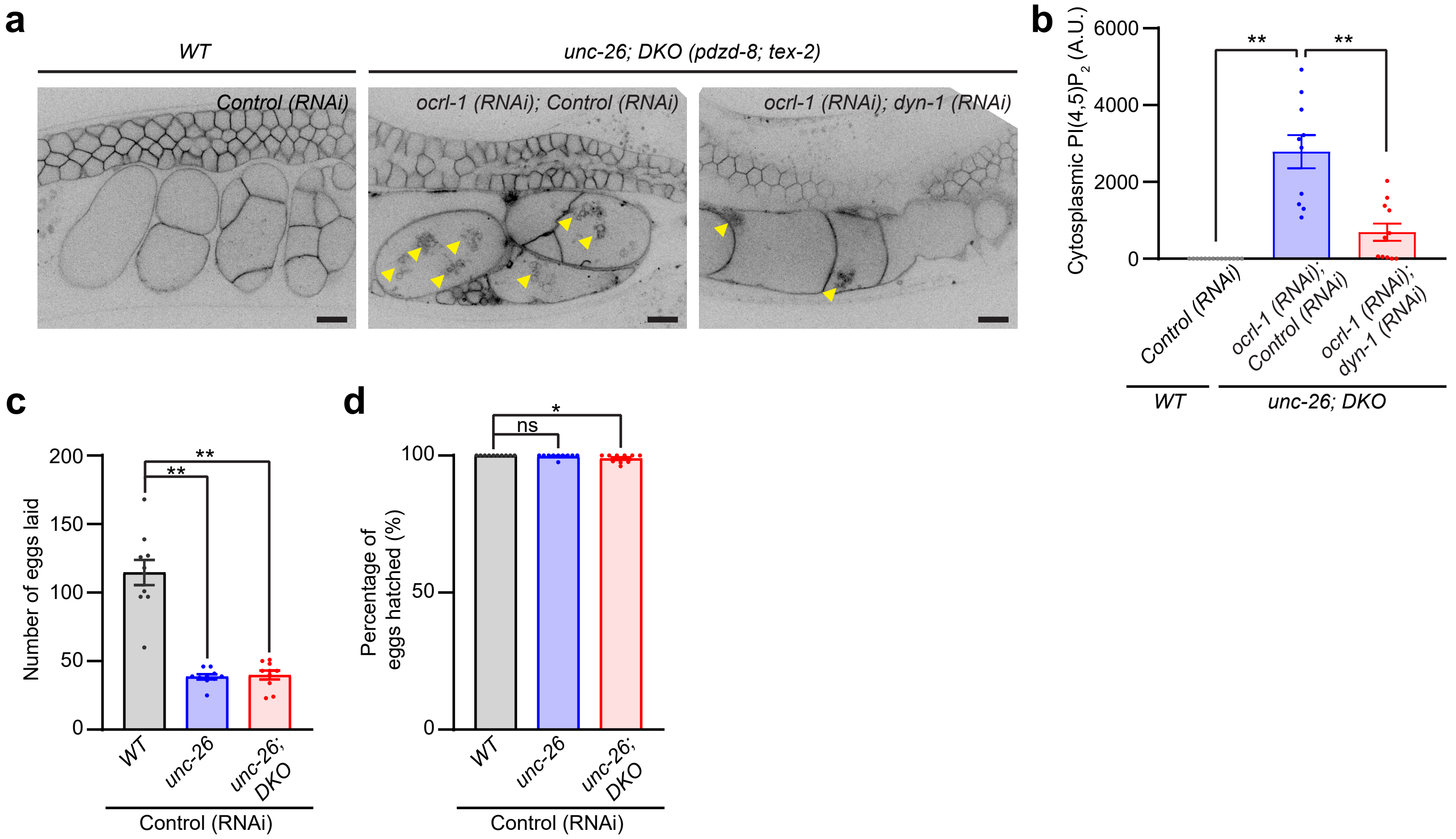

Supplementary Fig. 5 Cytoplasmic PI(4,5)P_2 vesicles in the absence of PDZD-8, TEX-2, UNC-26, and OCRL-1 originate from the plasma membrane through dynamin-dependent endycytosis. a Representative live spinning disc confocal (SDC) images of the uterus region of control RNAi-treated wild-type control (*WT*), OCRL-1 RNAi; control RNAi-treated *unc-26; double knock-out* [*DKO (pdzd-8; tex-2)*] mutants, and OCRL-1 RNAi; DYN-1 RNAi-treated *unc-26; DKO* mutants, expressing PI(4,5)P_2 biosensor (mCherry::PH$^{PLC\delta1}$). Yellow arrowheads indicate the clusters of PI(4,5)P_2-positive vesicles. Note the suppression of PI(4,5)P_2 accumulation in OCRL-1 RNAi; DYN-1 RNAi-treated *unc-26; DKO* mutants compared to OCRL-1 RNAi; control RNAi-treated *unc-26; DKO* mutants. Scale bars, 10µm. **b** Quantification of the cytoplasmic PI(4,5)P_2 levels (including PI(4,5)P_2-positive vesicles) in the uterus region of animals as shown in (a) [mean ± SEM, n=14 embryos (*WT*; control RNAi), n=10 embryos (*unc-26; DKO; ocrl-1* RNAi; control RNAi), n=11 embryos (*unc-26; DKO; ocrl-1* RNAi; *dyn-1* RNAi); Tukey's multiple comparisons test, **p<0.0001]. **c** Quantification of the number of eggs laid by control RNAi-treated wild-type control (*WT*) animals, control RNAi-treated *unc-26* mutants and control RNAi-treated *unc-26; double knock-out* [*DKO (pdzd-8; tex-2)*] [mean ± SEM, n=10 animals for all conditions; Dunnett's multiple comparisons test, **p<0.0001 (*unc-26*; Control RNAi), **p<0.0001 (*unc-26; DKO*; Control RNAi)]. **d** Quantification of the percentage of eggs successfully hatched in indicated conditions as in (a) [mean ± SEM, n=10 animals for all conditions; Dunnett's multiple comparisons test, *p=0.0335 (*unc-26; DKO*; Control RNAi), ns denotes not significant].

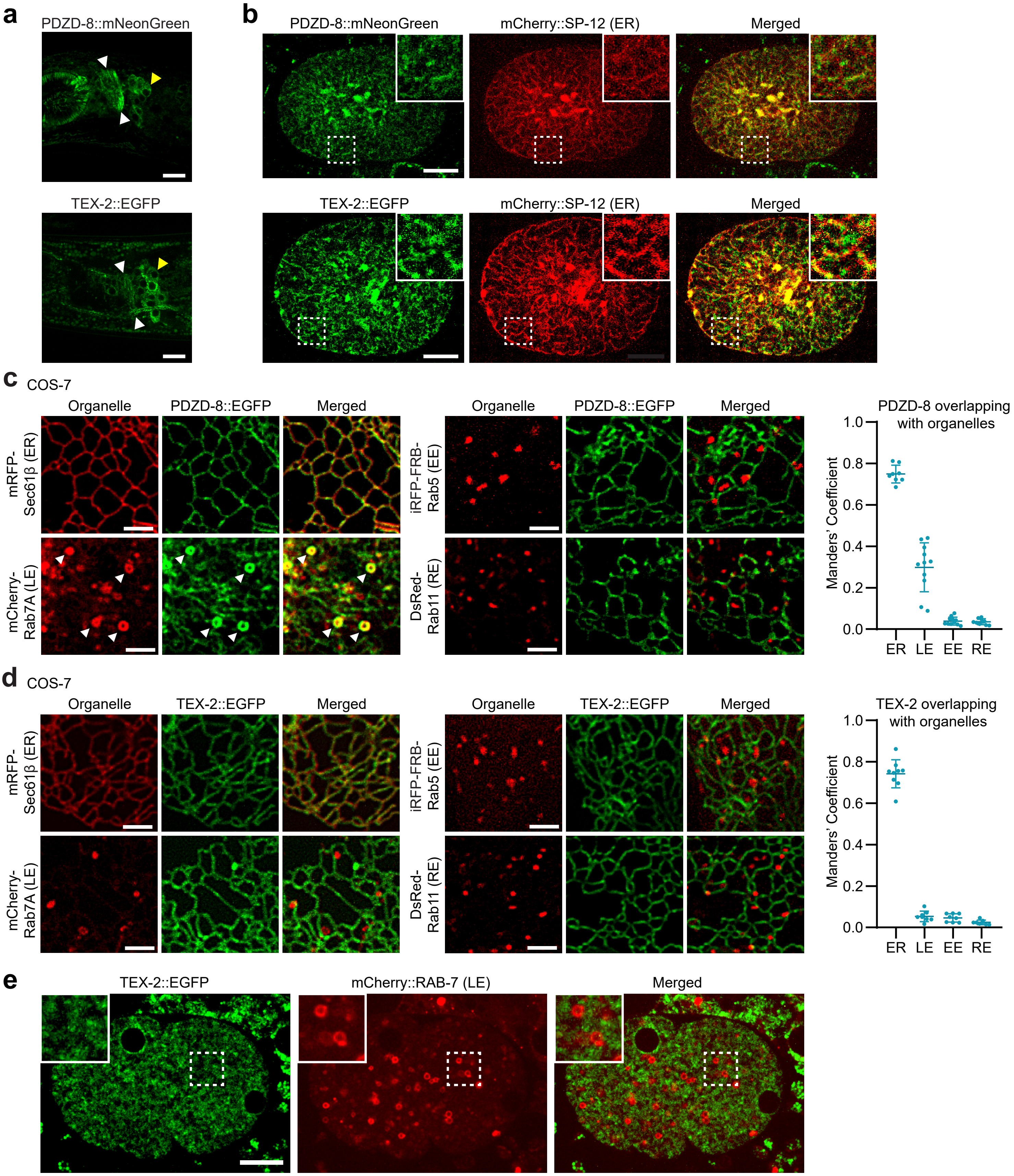
a
PDZD-8::mNeonGreen
TEX-2::EGFP
b
PDZD-8::mNeonGreen
mCherry::SP-12 (ER)
Merged
TEX-2::EGFP
mCherry::SP-12 (ER)
Merged
c
COS-7
Organelle
PDZD-8::EGFP
Merged
mRFP-Sec61β (ER)
mCherry-Rab7A (LE)
iRFP-FRB-Rab5 (EE)
DsRed-Rab11 (RE)
PDZD-8 overlapping with organelles
Manders' Coefficient
1.0
0.8
0.6
0.4
0.2
0.0
ER
LE
EE
RE
d
COS-7
Organelle
TEX-2::EGFP
Merged
mRFP-Sec61β (ER)
mCherry-Rab7A (LE)
iRFP-FRB-Rab5 (EE)
DsRed-Rab11 (RE)
TEX-2 overlapping with organelles
Manders' Coefficient
1.0
0.8
0.6
0.4
0.2
0.0
ER
LE
EE
RE
e
TEX-2::EGFP
mCherry::RAB-7 (LE)
Merged

Supplementary Fig. 6 Subcellular localization of PDZD-8 and TEX-2. a Representative live spinning disc confocal (SDC) images of the head regions of *C. elegans* expressing endogenous PDZD-8-tagged with mNeonGreen (PDZD-8::mNeonGreen) and endogenous TEX-2-tagged with EGFP (TEX-2::EGFP), as indicated. Scale bars, 10μm. **b** Representative live SDC images of equatorial planes of early embryos co-expressing either PDZD-8::mNeonGreen (top) or TEX-2::EGFP (bottom) and endoplasmic reticulum (ER) marker (mCherry::SP-12). The images are from polarity maintenance phase. Scale bar, 10μm. **c** Left: Representative live spinning disc confocal (SDC) structure illumination microscopy (SDC-SIM) images of COS-7 cells co-expressing *C. elegans* PDZD-8-tagged with EGFP (PDZD-8::EGFP) together with ER membrane marker (mRFP-Sec61β), late endosome (LE) marker (mCherry-Rab7A), early endosome (EE) marker (iRFP-FRB-Rab5) or recycling endosome (RE) marker (DsRed-Rab11) as indicated. Note the extensive co-localization of PDZD-8::EGFP with mRFP-Sec61β and the clustering of PDZD-8::EGFP around mCherry-Rab7A-positive LEs, indicated by white arrowheads. Scale bars, 2μm. Right: Quantification of the association of PDZD-8::EGFP with various organelles as shown in left [mean ± SD, n=8 cells (ER), n=11 cells (LE), n=11 cells (EE), n=10 cells (RE); Manders' coefficient]. **d** Left: Representative live SDC-SIM images of COS-7 cells co-expressing *C. elegans* TEX-2-tagged with EGFP (TEX-2::EGFP) together with ER membrane marker (mRFP-Sec61β), LE marker (mCherry-Rab7A), EE marker (iRFP-FRB-Rab5) or RE marker (DsRed-Rab11) as indicated. Note the extensive co-localization of TEX-2::EGFP with mRFP-Sec61 β. Scale bars, 2μm. Right: Quantification of the association of TEX-2::EGFP with various organelles as shown in left [mean ± SD, n=9 cells (ER), n=8 cells (LE), n=8 cells (EE), n=8 cells (RE); Manders' coefficient]. **e** A representative live SDC image of an equatorial plane of an early embryo from wild-type animals, co-expressing endogenously tagged TEX-2::EGFP and LE marker (mCherry::RAB-7). The image is from polarity establishment phase. Scale bar, 10μm.

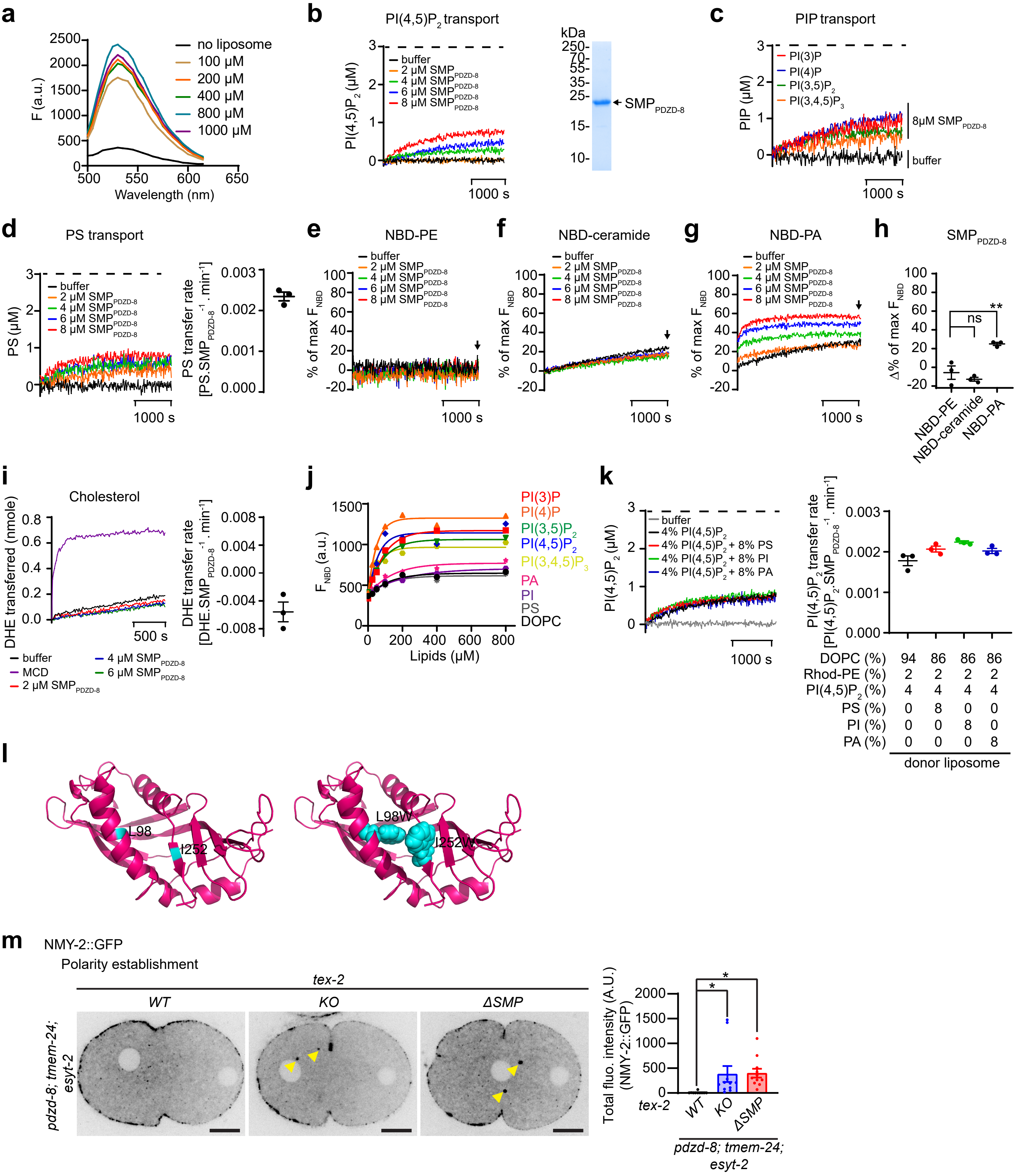
a
F (a.u.)
Wavelength (nm)
no liposome
100 μM
200 μM
400 μM
800 μM
1000 μM
b
PI(4,5)P2 transport
PI(4,5)P2 (μM)
buffer
2 μM SMPPDZD-8
4 μM SMPPDZD-8
6 μM SMPPDZD-8
8 μM SMPPDZD-8
1000 s
kDa
SMPPDZD-8
c
PIP transport
PIP (μM)
PI(3)P
PI(4)P
PI(3,5)P2
PI(3,4,5)P3
8μM SMPPDZD-8
buffer
1000 s
d
PS transport
PS (μM)
PS transfer rate [PS.SMPPDZD-8 -1 . min-1]
e
NBD-PE
f
NBD-ceramide
g
NBD-PA
% of max FNBD
h
SMPPDZD-8
Δ% of max FNBD
ns
**
NBD-PE
NBD-ceramide
NBD-PA
i
Cholesterol
DHE transferred (nmole)
DHE transfer rate [DHE.SMPPDZD-8 -1 . min-1]
500 s
buffer
MCD
2 μM SMPPDZD-8
4 μM SMPPDZD-8
6 μM SMPPDZD-8
j
FNBD (a.u.)
Lipids (μM)
PI(3)P
PI(4)P
PI(3,5)P2
PI(4,5)P2
PI(3,4,5)P3
PA
PI
PS
DOPC
k
PI(4,5)P2 (μM)
buffer
4% PI(4,5)P2
4% PI(4,5)P2 + 8% PS
4% PI(4,5)P2 + 8% PI
4% PI(4,5)P2 + 8% PA
1000 s
PI(4,5)P2 transfer rate [PI(4,5)P2.SMPPDZD-8 -1 . min-1]
DOPC (%) 94 86 86 86
Rhod-PE (%) 2 2 2 2
PI(4,5)P2 (%) 4 4 4 4
PS (%) 0 8 0 0
PI (%) 0 0 8 0
PA (%) 0 0 0 8
donor liposome
l
L98
I252
L98W
I252W
m
NMY-2::GFP
Polarity establishment
tex-2
WT
KO
ΔSMP
pdzd-8; tmem-24; esyt-2
Total fluo. intensity (A.U.) (NMY-2::GFP)
*
*
tex-2
WT
KO
ΔSMP
pdzd-8; tmem-24; esyt-2

Supplementary Fig. 7 Characterization of the lipid transport function of the SMP domain of PDZD-8. a Representative fluorescence spectra of NBD-PH_{FAPP} (0.5 μM) with increasing amount of liposomes containing 2% PI(4,5)P_2 [98% DOPC and 2% brain PI(4,5)P_2] (from 0 to 1000 μM total lipids). Note the gradual shift of the emission wavelength of NBD to a lower wavelength (i.e., a blue shift), consistent with the insertion of NBD moiety into liposomal membranes upon binding of NBD-PH_{FAPP} with PI(4,5)P_2[97]. **b** Left: Representative time course of PI(4,5)P_2 transfer from donor to acceptor liposomes by increasing amount of SMP_{PDZD-8} as indicated. The dashed line corresponds to the condition that mimics full PI(4,5)P_2 equilibration between donor and acceptor liposomes. Note the gradual increase of PI(4,5)P_2 transfer with increasing amount of SMP_{PDZD-8} in solution. Right: A representative SDS-PAGE gel image of purified SMP_{PDZD-8} proteins used in the assay. **c** Representative time course of PIP transfer [PI(3)P, PI(4)P, PI(3,5)P_2 and PI(3,4,5)P_3] from donor to acceptor liposomes by purified SMP_{PDZD-8} proteins (8 μM). The dashed line corresponds to the condition that mimics full PIP equilibration between donor and acceptor liposomes. **d** Left: Representative time course of phosphatidylserine (PS) transfer from donor to acceptor liposomes by increasing amount of SMP_{PDZD-8} as indicated. Donor liposomes [4% PS, 2% Rhodamine-PE, 94% DOPC] and acceptor liposomes [100% DOPC] (0.6 mM total lipids) were incubated with purified NBD-$C2_{Lact}$ proteins (0.5 μM) and purified SMP_{PDZD-8} proteins. The dashed line corresponds to the condition that mimics full PS equilibration between donor and acceptor liposomes. Note the gradual increase of PS transfer with increasing amount of SMP_{PDZD-8} in solution. Right: Quantification of PS transport rates of SMP_{PDZD-8} (8 μM) [mean ± SEM, n=3 independent experiments]. **e-g** Representative time course of NBD-labelled lipid transfer from donor [4% NBD-labelled lipids (either one of PE, ceramide, or PA as indicated), 2% Rhodamine-PE, 94% DOPC] to acceptor [100% DOPC] liposomes (0.6 mM total lipids) by increasing amount of SMP_{PDZD-8} as indicated. **h** The difference (Δ) of the percentage of max NBD fluorescence [obtained by the addition of N-Dodecyl β-D-maltoside (DDM) at the end of each transfer reaction, see Methods] between buffer alone and buffer containing SMP_{PDZD-8} (8 μ M), corresponding to the end of the experiment as indicated by the arrow as shown in (e-g) [mean ± SEM, n=3 independent experiments for each condition; Dunnett's multiple comparisons test, **p=0.0047 (NBD-PE vs. NBD-PA); ns denotes not significant]. **i** Left: Representative time course of DHE transfer from donor (10% DHE and 90% DOPC) to acceptor (2.5% DNS-PE and 97.5% DOPC) liposomes (0.6 mM total lipids) by increasing amount of SMP_{PDZD-8} as indicated. Methyl-β-cyclodextrin (MCD) (1 mM) was used to determine DHE equilibration. A series of liposomes with different DHE mol% were prepared to plot a calibration curve to convert FRET signals to DHE molecules transferred (see Methods). Right: Quantification of DHE transport rates of SMP_{PDZD-8} (6 μM) [mean ± SEM, n=3 independent experiments]. **j** Representative NBD fluorescence intensity measured at 530 nm for different membrane compositions (100% DOPC vs. 98% DOPC and 2% of indicated lipid) as function of total lipid concentration. Note the progressive increase in the intensity of NBD fluorescence as NBD-PH_{FAPP} (0.5 μM) bound to increasing amount of liposomes containing PIPs as indicated. **k** Left: Representative time course of PI(4,5)P_2 transfer from donor liposomes [4% PI(4,5)P_2, 2% Rhodamine-PE, 94% DOPC] or donor liposomes that additionally contains 8% of anionic lipids [4% PI(4,5)P_2, 2% Rhodamine-PE, 8% anionic lipid (either one of PS, PA, or PI as indicated), 86% DOPC] to acceptor liposomes [100% DOPC] by purified SMP_{PDZD-8} proteins (8 μM). The dashed line corresponds to the condition that mimics full PI(4,5)P_2 equilibration between donor and acceptor liposomes. Right: Quantification of PI(4,5)P_2 transport rates of SMP_{PDZD-8} (8 μM) [mean ± SEM, n=3 independent experiments for each condition]. **l** Ribbon diagram of (left) wild-type SMP_{PDZD-8} and (right) mutant SMP_{PDZD-8} with L98W and I252W mutations. Note that the bulky tryptophan (W) side chains in blue in mutant PDZD-8 SMP domain are predicted to hinder the insertion or binding of lipids. **m** Left: Representative live SDC images of equatorial planes of early embryos from *pdzd-8; tmem-24; esyt-2* mutants carrying indicated alleles of *tex-2*, expressing NMY-2-tagged with GFP (NMY-2::GFP). Images are from polarity establishment phase. Scale bars, 10μm. Right: Quantification of the total fluorescence intensity of NMY-2::GFP puncta per minute during polarity establishment of the early embryogenesis [mean ± SEM, n=10 embryos (*tex-2 WT*), n=11 embryos (*tex-2 KO*), n=11 embryos (*tex-2 ΔSMP*); Dunnett's multiple comparisons test, *p=0.0414 (*tex-2 KO*), *p=0.0320 (*tex-2 ΔSMP*)].

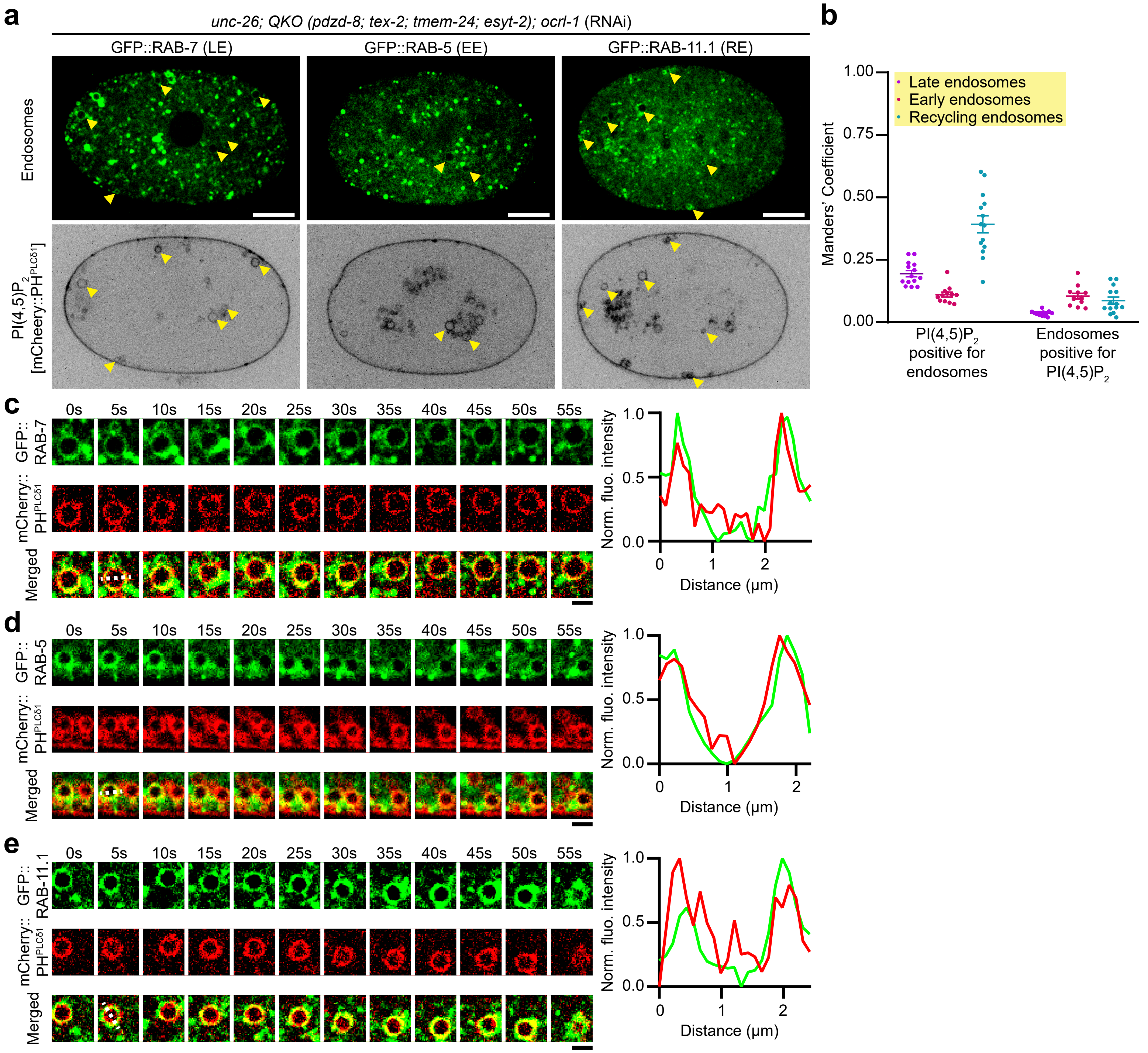

Supplementary Fig. 8 Massive accumulation of PI(4,5)P_2 occurs throughout the endosomal system in the absence of SMP proteins and PI(4,5)P_2 phosphatases. a Representative live spinning disc confocal (SDC) images of equatorial planes of early embryos from OCRL-1 RNAi-treated *unc-26; quadruple knock-out* [*QKO (pdzd-8; tex-2; tmem-24; esyt-2)*] mutants co-expressing PI(4,5)P_2 biosensor (mCherry::$PH^{PLC\delta1}$) with endosomal markers, as indicated. Yellow arrowheads indicate PI(4,5)P_2-positive vesicles that are decorated and endosomal markers. Scale bars, 10µm. LE, EE and RE denote late endosome, early endosome and recycling endosome, respectively. **b** Quantification of the co-localization of PI(4,5)P_2-positive vesicles with endosomes as indicated. [mean ± SEM, n=14 cells (GFP::RAB-7), n=12 cells (GFP::RAB-5), n=14 cells (GFP::RAB-11.1); Manders' coefficient]. **c-e** Left: Time-lapse SDC images over the period of 55 seconds of representative PI(4,5)P_2-positive vesicles from OCRL-1 RNAi-treated *unc-26; QKO* mutants, expressing mCherry::$PH^{PLC\delta1}$ together with either (c) GFP::RAB-7, (d) GFP::RAB-5, or (e) GFP::RAB-11.1. Note the extensive association of PI(4,5)P_2-positive vesicles with GFP::RAB-7 and GFP::RAB-11.1 and the transient association of a PI(4,5)P_2-positive vesicle with GFP::RAB-5. Scale bars, 2µm. Right: Line scan profiles of mCherry::$PH^{PLC\delta1}$ (red) and either (c) GFP::RAB-7, (d) GFP::RAB-5, or (e) GFP::RAB-11.1 (green) fluorescence signals along the white dotted lines as indicated.

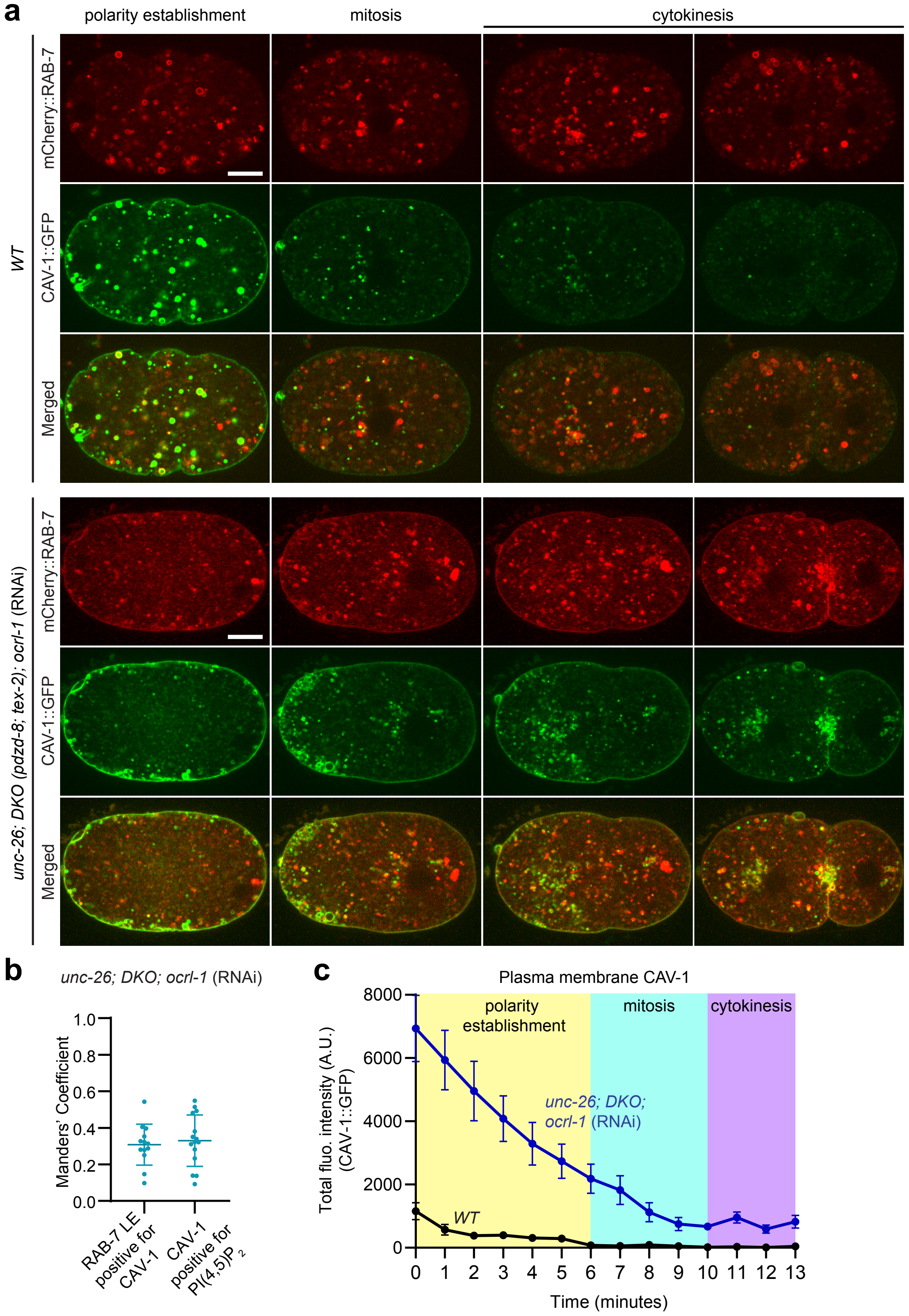

Supplementary Fig. 9 Simultaneous depletion PDZD-8, TEX-2, and PI(4,5)P_2 phosphatases disrupts the degradative capacity of endosomes. a Representative live spinning disc confocal (SDC) images of equatorial planes of early embryos from wild-type control (*WT*) and OCRL-1 RNAi-treated *unc-26; double knock-out* [*DKO (pdzd-8; tex-2)*] mutants, co-expressing the late endosome marker [endogenous RAB-7-tagged with mCherry (mCherry::RAB-7)] and CAV-1-tagged with GFP (CAV-1::GFP). Images of each row are from time-lapse movies of the same embryos at different phases as indicated. Scale bars, 10μm. **b** Quantification of the association of CAV-1::GFP with either RAB-7-positive late endosomes (LEs) or PI(4,5)P_2-positive vesicles in OCRL-1 RNAi-treated *unc-26; DKO* mutants as indicated [mean ± SD, n=13 embryos (RAB-7 LE), n=15 embryos (PI(4,5)P_2); Manders' coefficient]. **c** Time-course analysis of the total fluorescence intensity of plasma membrane CAV-1::GFP during polarity establishment (0-6 minutes), mitosis (6-10 minutes), and cytokinesis (10-13 minutes) phases for *WT* and OCRL-1 RNAi-treated *unc-26; DKO* mutants early embryos as shown in Fig. 8a [mean ± SEM, n=10 embryos (*WT*), n=9 embryos (*unc-26; DKO; ocrl-1* RNAi)].

Supplementary Table 1

REAGENT or RESOURCE	SOURCE	IDENTIFIER
Bacterial and Virus Strains		
E. coli OP50	Caenorhabditis Genetics Center	https://cgc.umn.edu/strain/OP50
E. coli HT115 (DE3)	Caenorhabditis Genetics Center	https://cgc.umn.edu/strain/HT115(DE3)
NEB Turbo Competent *E. Coli*	NEB	C2984I
E. coli BL21-DE3 Rosetta	NTU PPP	
Chemicals, Peptides, and Recombinant Proteins		
LysoTracker™ Red DND-99	Life Technologies	L7528
MitoTracker™ Red CMXRos	Life Technologies	M7512
Q5 High-Fidelity DNA Polymerase	NEB	M0491S
Proteinase K	Sigma-Aldrich	P4850
1,2-dioleoyl-sn-glycero-3-phosphocholine (DOPC)	Avanti Polar Lipids	850375
L-α-phosphatidylinositol-4,5-bisphosphate (Brain, Porcine) (ammonium salt) (brain PI(4,5)P_2)	Avanti Polar Lipids	840046
1,2-dipalmitoyl-sn-glycero-3-phosphoethanolamine-N-(lissamine rhodamine B sulfonyl) (ammonium salt) (16:0 Liss Rhod PE)	Avanti Polar Lipids	810158
1,2-dipalmitoyl-sn-glycero-3-phosphoethanolamine-N-(7-nitro-2-1,3-benzoxadiazol-4-yl) (ammonium salt) 16:0 NBD PE	Avanti Polar Lipids	810144
L-α-phosphatidylinositol-4-phosphate (Brain, Porcine) (ammonium Salt) Brain PI(4)P	Avanti Polar Lipids	840045
1,2-dioleoyl-sn-glycero-3-phospho-(1'-myo-inositol-3'-phosphate) (ammonium salt) 18:1 PI(3)P	Avanti Polar Lipids	850150
1,2-dioleoyl-sn-glycero-3-phospho-(1'-myo-inositol-3',5'-bisphosphate) (ammonium salt) 18:1 PI(3,5)P_2	Avanti Polar Lipids	850154
1,2-dioleoyl-sn-glycero-3-phospho-(1'-myo-inositol-3',4',5'-trisphosphate) (ammonium salt) 18:1 PI(3,4,5)P_3	Avanti Polar Lipids	850156
1-oleoyl-2-{12-[(7-nitro-2-1,3-benzoxadiazol-4-yl)amino]dodecanoyl}-sn-glycero-3-phosphate (ammonium salt) 18:1-12:0 NBD PA	Avanti Polar Lipids	810176
N-[12-[(7-nitro-2-1,3-benzoxadiazol-4-yl)amino]dodecanoyl]-D-erythro-sphingosine C12-NBD Ceramide	Avanti Polar Lipids	810211
ergosta-5,7,9(11),22-tetraen-3ß-ol dehydroergosterol (DHE)	Avanti Polar Lipids	810253
1,2-dioleoyl-sn-glycero-3-phosphoethanolamine-N-(5-dimethylamino-1-naphthalenesulfonyl) (ammonium salt) 18:1 Dansyl PE	Avanti Polar Lipids	810330
L-α-phosphatidylserine (Brain, Porcine) (sodium salt) Brain PS	Avanti Polar Lipids	840032
L-α-phosphatidylinositol (Liver, Bovine) (sodium salt) Liver PI	Avanti Polar Lipids	840042
1,2-dioleoyl-sn-glycero-3-phosphate (sodium salt) 18:1 PA	Avanti Polar Lipids	840875
n-dodecyl-ß-D-maltoside (DDM)	Avanti Polar Lipids	850520
Lysozyme from chicken egg white	Sigma-Aldrich	62970
DNase	Sigma-Aldrich	DN25
Tris (2-carboxyethyl) phosphine (TCEP)	Sigma-Aldrich	C4706
Isopropyl-1-thio-B-d-galactopyranoside (IPTG)	ThermoFisher	R0392
COMPLETE(TM), EDTA-FREE PROTEASE INHIBI&	Merck	11873580001
HisPur™ Ni-NTA Resin	ThermoFisher	88222
IANBD-amide	Invitrogen	D2004
Critical Commercial Assays		
Q5® Site-Directed Mutagenesis Kit	New England	E0554S
Experimental Models: Cell Lines		
COS-7 cells	Min Wu Lab	YS72

Experimental Models: Organisms/Strains		
C. elegans: Strain OD70: *unc-119(ed3) III; ltIs44[pie-1p::mCherry::PH(PLC1delta1) + unc-119(+)]*	Caenorhabditis Genetics Center	OD70; WormBase: WBStrain00029215
C. elegans: Strain SAH346: *F55C12.5(syb670) II ; esyt-2(syb709) III; C53B4.4(syb664) IV; R11G1.6(tm10626) X; ltIs44[pie-1p::mCherry::PH(PLC1delta1) + unc-119(+)]*	Cross - this paper	SAH346
C. elegans: Strain SAH394: *F55C12.5(syb670) II; esyt-2(syb709) III; C53B4.4(syb664) IV; zuIs45[nmy-2::NMY-2::GFP + unc-119(+)] V; R11G1.6(tm10626) X; ltIs44[pie-1p::mCherry::PH(PLC1delta1) + unc-119(+)]*	Cross - this paper	SAH394
C. elegans: Strain PHX1716: *F55C12.5(syb670) II; esyt-2(syb709) ani-1[syb1710(wrmScarlet::ANI-1)] III; C53B4.4(syb664) IV; zuIs45[nmy-2::NMY-2::GFP + unc-119(+)] ; R11G1.6(tm10626) X*	SunyBiotech - this paper	PHX1716
C. elegans: Strain SAH393: *F55C12.5(syb670) II ; esyt-2(syb709) III; C53B4.4(syb664) IV; zuIs45[nmy-2::NMY-2::GFP + unc-119(+)] V; R11G1.6(tm10626) X; zbIs2(pie-1::lifeACT::RFP)*	Cross - this paper	SAH393
C. elegans: Strain SAH468: *F55C12.5(syb670) II; esyt-2(syb709) ani-1[syb1710(wrmScarlet::ANI-1)] III; C53B4.4(syb664) IV; R11G1.6(tm10626) X; ojIs35[pie-1::GFP::rab-11.1 + unc-119(+)]*	Cross - this paper	SAH468
C. elegans: Strain SAH416: *F55C12.5(syb670) II; esyt-2(syb709) ani-1[syb1710(wrmScarlet::ANI-1)] III; C53B4.4(syb664) IV; R11G1.6(tm10626) X; weIs15[pie-1p::GFP::eea-1(FYVEx2) + unc-119(+)]*	Cross - this paper	SAH416
C. elegans: Strain SAH477: *sybIs2380 [pie-1p::GFP::rab-7 + unc-119(+)] I; F55C12.5(syb670) II; esyt-2(syb709) ani-1[syb1710(wrmScarlet::ANI-1)] III; C53B4.4(syb664) IV; R11G1.6(tm10626) X*	Cross - this paper	SAH477
C. elegans: Strain JJ1473: *unc-119(ed3) III; zuIs45[nmy-2::NMY-2::GFP + unc-119(+)] V*	Caenorhabditis Genetics Center	JJ1473; WormBase: WBStrain00022491
C. elegans: Strain SAH347: *F55C12.5(syb670) II; esyt-2(syb709) III; C53B4.4(syb664) IV; zuIs45[nmy-2::NMY-2::GFP + unc-119(+)] V; R11G1.6(tm10626) X*	Cross - this paper	SAH347
C. elegans: Strain SAH308: *F55C12.5(syb670) II; C53B4.4(syb664) IV; zuIs45[nmy-2::NMY-2::GFP + unc-119(+)] V*	Cross - this paper	SAH308
C. elegans: Strain SAH415: *F55C12.5(syb670) II; esyt-2(syb709) III; zuIs45[nmy-2::NMY-2::GFP + unc-119(+)] V; R11G1.6(tm10626) X*	Cross - this paper	SAH415
C. elegans: Strain SAH366: *F55C12.5(syb670) II; C53B4.4(syb664) IV; zuIs45[nmy-2::NMY-2::GFP + unc-119(+)] V; R11G1.6(tm10626) X*	Cross - this paper	SAH366
C. elegans: Strain SAH335: *esyt-2(syb709) III; C53B4.4(syb664) IV; zuIs45[nmy-2::NMY-2::GFP + unc-119(+)] V; R11G1.6(tm10626) X*	Cross - this paper	SAH335
C. elegans: Strain SAH325: *F55C12.5(syb670) II; esyt-2(syb709) II; C53B4.4(syb664) IV; zuIs45[nmy-2::NMY-2::GFP + unc-119(+)] V*	Cross - this paper	SAH325
C. elegans: Strain SAH311: *esyt-2(syb709) III; R11G1.6(tm10626) X; zuIs45[nmy-2::NMY-2::GFP + unc-119(+)] V*	Cross - this paper	SAH311
C. elegans: Strain SAH258: *C53B4.4(syb664) IV; zuIs45[nmy-2::NMY-2::GFP + unc-119(+)] V*	Cross - this paper	SAH258
C. elegans: Strain SAH250: *R11G1.6(tm10626) X; zuIs45[nmy-2::NMY-2::GFP + unc-119(+)] V*	Cross - this paper	SAH250
C. elegans: Strain SAH241: *esyt-2(syb709) III; zuIs45[nmy-2::NMY-2::GFP + unc-119(+)] V*	Cross - this paper	SAH241
C. elegans: Strain SAH264: *F55C12.5(syb670) II; zuIs45[nmy-2::NMY-2::GFP + unc-119(+)] V*	Cross - this paper	SAH264
C. elegans: Strain SAH420: *unc-26(s1710) IV; zuIs45[nmy-2::NMY-2::GFP + unc-119(+)] V; ltIs44[pie-1p::mCherry::PH(PLC1delta1) + unc-119(+)]*	Cross - this paper	SAH420
C. elegans: Strain SAH426: *F55C12.5(syb670) II; C53B4.4(syb664) unc-26(s1710) IV; zuIs45[nmy-2::NMY-2::GFP + unc-119(+)] V; ltIs44[pie-1p::mCherry::PH(PLC1delta1) + unc-119(+)]*	Cross - this paper	SAH426
C. elegans: Strain SAH421: *zuIs45[nmy-2::NMY-2::GFP + unc-119(+)] V; ltIs44[pie-1p::mCherry::PH(PLC1delta1) + unc-119(+)]*	Cross - this paper	SAH421
C. elegans: Strain SAH427: *F55C12.5(syb670) II; esyt-2(syb709) III; C53B4.4(syb664) unc-26(s1710) IV; zuIs45[nmy-2::NMY-2::GFP + unc-119(+)] V; R11G1.6(tm10626) X; ltIs44[pie-1p::mCherry::PH(PLC1delta1) + unc-119(+)]*	Cross - this paper	SAH427

C. elegans: Strain SAH564: *ani-1[syb1710(wrmScarlet::ANI-1)] III; unc-26(s1710) IV*	Cross - this paper	SAH564
C. elegans: Strain SAH524: *F55C12.5(syb670) II; esyt-2(syb709) ani-1[syb1710(wrmScarlet::ANI-1)] III; C53B4.4(syb664) IV; unc-26(s1710) IV; R11G1.6(tm10626) X*	Cross - this paper	SAH524
C. elegans: Strain PHX2977: *F55C12.5(syb670) II; esyt-2(syb709) III; C53B4.4(syb2977) IV; zuIs45[nmy-2::NMY-2::GFP+unc-119(+)] V; R11G1.6(tm10626) X*	SunyBiotech - this paper	PHX2977
C. elegans: Strain PHX3276: *F55C12.5(syb670) II; esyt-2(syb709) III; C53B4.4(syb3053 syb3276) IV; zuIs45[nmy-2::NMY-2::GFP + unc-119(+)] V; R11G1.6(tm10626) X*	SunyBiotech - this paper	PHX3276
C. elegans: Strain SAH561: *F55C12.5(syb2164) II; esyt-2(syb709) III; C53B4.4(syb2977) IV; zuIs45[nmy-2::NMY-2::GFP+unc-119(+)] V; R11G1.6(tm10626) X*	Cross - this paper	SAH561
C. elegans: Strain PHX2164: *F55C12.5(syb2164) II; esyt-2(syb709) III; C53B4.4(syb664) IV; zuIs45 [nmy-2::NMY-2::GFP + unc-119(+)] V; R11G1.6(tm10626) X*	SunyBiotech - this paper	PHX2164
C. elegans: Strain SAH563: *F55C12.5(yas46[F55C12.5::EGFP^3xFLAG]) II; ocfIs2[pie-1p:mCherry::sp12::pie-1 3'UTR + unc-119(+)]*	Cross - this paper	SAH563
C. elegans: Strain SAH665: *F55C12.5(syb670) II; esyt-2(syb709) III; C53B4.4(syb664) unc-26(s1710) IV; R11G1.6(tm10626) X; ltIs44[pie-1p::mCherry::PH(PLC1delta1) + unc-119(+)]; pwIs20 [pie-1p::GFP::rab-5 + unc-119(+)]*	Cross - this paper	SAH665
C. elegans: Strain SAH542: *sybIs2380 [pie-1p::GFP::rab-7 + unc-119(+)] I; F55C12.5(syb670) II; esyt-2(syb709) III; C53B4.4(syb664) unc-26(s1710) IV; R11G1.6(tm10626) X; ltIs44[pie-1p::mCherry::PH(PLC1delta1) + unc-119(+)]*	Cross - this paper	SAH542
C. elegans: Strain SAH558: *F55C12.5(syb670) II; esyt-2(syb709) III; C53B4.4(syb664) unc-26(s1710) IV; R11G1.6(tm10626) X; ltIs44[pie-1p::mCherry::PH(PLC1delta1) + unc-119(+)]; ojIs35 [pie-1::GFP::rab-11.1 + unc-119(+)]*	Cross - this paper	SAH558
C. elegans: Strain SAH634*: rab-7(syb4141[mCherry::RAB-7) II; unc-119(ed3) III; ojIs23 [pie-1p::GFP::C34B2.10]*	Cross - this paper	SAH634
C. elegans: Strain SAH607*: C53B4.4(syb4099[C53B4.4::mNeongreen]) IV; rab-7(syb4141[mCherry::RAB-7]) II*	Cross - this paper	SAH607
C. elegans: Strain SAH631*: pwIs28[pie-1p::cav-1::GFP(7) + unc-119(+)]; ltIs44[pie-1p::mCherry::PH(PLC1delta1) + unc-119(+)]*	Cross - this paper	SAH631
C. elegans: Strain SAH587*: F55C12.5(syb670) II; C53B4.4(syb664) unc-26(s1710) IV; pwIs28[pie-1p::cav-1::GFP(7) + unc-119(+)]; ltIs44[pie-1p::mCherry::PH(PLC1delta1) + unc-119(+)]*	Cross - this paper	SAH587
C. elegans: Strain SAH655: *F55C12.5(syb670) rab-7(syb4141[mCherry::RAB-7]) II ; C53B4.4(syb664) unc-26(s1710) IV; pwIs28[pie-1p::cav-1::GFP(7) + unc-119(+)]*	Cross - this paper	SAH655
C. elegans: Strain SAH630*: ppk-1(syb4109[PPK-1::EGFP) I*; *ltIs44[pie-1p::mCherry::PH(PLC1delta1) + unc-119(+)]*	Cross - this paper	SAH630
C. elegans: Strain SAH614*: ppk-1(syb4109[PPK-1::EGFP) I; F55C12.5(syb670) II ; esyt-2(syb709) III; C53B4.4(syb664) IV; R11G1.6(tm10626) X; ltIs44[pie-1p::mCherry::PH(PLC1delta1) + unc-119(+)]*	Cross - this paper	SAH614
C. elegans: Strain SAH638*: C53B4.4(syb4099[C53B4.4::mNeongreen]) IV; ocfIs2[pie-1p:mCherry::sp12::pie-1 3'UTR + unc-119(+)]*	Cross - this paper	SAH638
C. elegans: Strain SAH633*: F55C12.5(yas46[F55C12.5::EGFP^3xFLAG]) rab-7(syb4141[mCherry::RAB-7]) II*	Cross - this paper	SAH633
C. elegans: Strain SAH617: *rab-7(syb4141[mCherry::RAB-7]) II; pwIs28[pie-1p::cav-1::GFP(7) + unc-119(+)]*	Cross - this paper	SAH617
Oligonucleotides		
TATTGTATCCCATGTGGAGTGTTTTAGAGCTAGAAATAGCAAGT	This paper	F55C12.5_GUIDE-5_FWD
CAAGACATCTCGCAATAGG	This paper	CAS9_MUT_REV
ACGTTGTAAAACGACGGCCAGTCGCCGGCAGCCAAATTACAGGCTGTCGCGAGGGTGAGA	This paper	F55C12.5_C_282_5' FW
ATCGATGCTCCTGAGGCTCCCGATGCTCCCTTGTTGTACCCCATATGGAGCAGGGGATT	This paper	F55C12.5_C_5'_REV
GTGATTACAAGGATGACGATGACAAGAGATAAACAGACTATTCAAGTCTTTTTTCACAA	This paper	F55C12.5_C_3'_FWD
GGAAACAGCTATGACCATGTTATCGATTTCGGCTTTCGTACGTGTTGCTGTACAACCGAT	This paper	F55C12.5_C_3'_REV

AGCTCAAGCTTCGCCACCATGTTAATAGCATTTCTAGTCGGG	This paper	5'_HINDIII_CEPDZD8_NS
GGCCCGCGGTACCCGGGAATGGGCTTCAACTGCATCCG	This paper	3'_KPNI_CEPDZD8_CAS
GCTTCGAATTCGCCACCATGAGTCGACTAAGTCTTGGAGGC	This paper	5'_ECORI_CETEX2_NS
GCCCGCGGTACCCGCTTATTGTATCCCATGTGGAGTAGG	This paper	3'_KPNI_CETEX2_CAS
GAACCTGTACTTCCAATCCATGGGTGATGGACAAGGAATTTCAAA	This paper	5'NCOI_PDZD8_VC02
CGGAGCTCGAATTCGGATCCCTATGAAGCCTGGAATATTGGATTC	This paper	3'BAMHI_PDZD8_VC03
CTTCCAATCCATGGAAGGTGTGCTGTATAAGTGGACTAAC	This paper	5'NCOI_PH-FAPP
ATTCGGATCCTCAAGTACGCGTATCCGTAAGACTAGCTTT	This paper	3'BAMHI_PH-FAPP
GAACCTGTACTTCCAATCCATGGAAGGTGTGCTGTATAAGTGGACTAACTACCTTTGCGGATGGCAACCGCGCTGGTTTGTACTGGATAATGGAATCTTATCATATTATGACTCTCAGGACGACGTTTCAAAAGGCTCCAAGGGCTCTATTAAAATGGCAGTTTGTGAAATCAAAGTCCACAGCGCTGATAACACTCGTATGGAACTTATTATCCCCGGAGAGCAGCACTTTTACATGAAGGCTGTAAACGCTGCTGAGCGTCAGCGCTGGCTTGTGGCGCTGGGAAGTAGCAAAGCTAGTCTTACGGATACGCGTACTTGAGGATCCGAATTCGAGCTC	This paper	PH-FAPP
TACTTCCAATCCATGGGGGCGACAGAACCGCTTGGGCTGA	This paper	5'NcoI_Lact-C2_w_mutations
TCGAATTCGGATCCTCAAGCACCAAGTAATTCAACGCGAA	This paper	3'BamHI_Lact-C2_w_mutations
tacttccaatccATGGGGgcgACAGAACCGCTTGGGCTGAAGGACAACACGATTCCGAACAAACAGATTACGGCGTCGTCTTATTACAAAACATGGGGTCTTTCCGCTTTTTCGTGGTTCCCATACTATGCGCGTTTAGATAATCAAGGTAAGTTCAATGCATGGACTGCGCAGACTAACTCCGCGTCGGAATGGTTACAAATTGACTTGGGGAGCCAGAAACGTGTTACGGGTATTATCACACAAGGTGCCCGCGATTTCGGAtgtATTCAATACGTCGCTGCGTATCGCGTCGCTTACGGAGATGATGGCGTTACTTGGACGGAGTACAAAGACCCCGGGGCGAGCGAGAGCAAAATCTTTCCCGGTAACATGGATAACAACAGCCATAAGAAGAACATCTTCGAGACGCCGTTCCAAGCTCGCTTCGTTCGCATTCAGCCAGTCGCTTGGCATAATCGTATTACACTTCGCGTTGAATTACTTGGTgctTGAGGATCCGAATTCGA	This paper	Lact-C2 (C270A_C427A_H352C)
Recombinant DNA		
L4440 (Control RNAi)	Addgene	1654
OCRL-1 RNAi	Source Bioscience (Ahringer Library)	I-4J06
DYN-1 RNAi	Source Bioscience (Ahringer Library)	X-7K09
pDD162_F55C12.5_gRNA	This paper	DJ53
pDD282_F55C12.5_GFP	This paper	DJ44
pCFJ352_GFP_RAB-7	This paper	YS16
pCePDZD8-EGFP	This paper	TN216
pCeTEX-2-EGFP	This paper	TN215
mCh-Rab7A	Addgene	61804
DsRed-rab11 WT	Addgene	12679
iRFP-FRB-Rab5	Addgene	51612
RFP-Sec61β	De Camilli Lab	YS134
pNIC28-Bsa4 His-SMP-PDZD8 vc03	This paper	BE108
pNIC28-Bsa4 PH-FAPP (T13C/C37S/C94S)	This paper	BE103
pNIC-Bsa4 Lact-C2 (C270A/C427A/H352C)	This paper	BE171
Software and Algorithms		

Fiji (Image J) Version 1.53c	Schneider et al., 2012	https://imagej.nih.gov/ij/
Prism Version 8.0.1	GraphPad	https://www.graphpad.com/scientific-software/prism/
I-TASSER	Zhang., 2008	https://zhanglab.ccmb.med.umich.edu/I-TASSER/
PyMOL Version 2.2.0	DeLano., 2002	https://pymol.org/2/
Other		
Polystyrene beads (15.6 ± 0.03 μm diameter)	Bangs Labs	NT29N
100 nm NanoSizer Extruder	T&T Scientific	TT-002-0001
Liposomes used in lipid transfer assays		
$PI(4,5)P_2$ transfer<br>L_D: (4% brain $PI(4,5)P_2$, 2% Rhod-PE, 94% DOPC)	This paper	
$PI(4,5)P_2$ transfer<br>L_A: (100% DOPC)	This paper	
$PI(4,5)P_2$ transfer – equilibrium condition<br>L_D: (2% brain $PI(4,5)P_2$, 2% Rhod-PE, 96% DOPC)	This paper	
$PI(4,5)P_2$ transfer – equilibrium condition<br>L_A: (2% brain $PI(4,5)P_2$, 98% DOPC)	This paper	
PI(3)P transfer<br>L_D: (4% PI(3)P, 2% Rhod-PE, 94% DOPC)	This paper	
PI(3)P transfer<br>L_A: (100% DOPC)	This paper	
PI(3)P transfer – equilibrium condition<br>L_D: (2% PI(3)P, 2% Rhod-PE, 96% DOPC)	This paper	
PI(3)P transfer – equilibrium condition<br>L_A: (2% PI(3)P, 98% DOPC)	This paper	
PI(4)P transfer<br>L_D: (4% brain PI(4)P, 2% Rhod-PE, 94% DOPC)	This paper	
PI(4)P transfer<br>L_A: (100% DOPC)	This paper	
PI(4)P transfer – equilibrium condition<br>L_D: (2% brain PI(4)P, 2% Rhod-PE, 96% DOPC)	This paper	
PI(4)P transfer – equilibrium condition<br>L_A: (2% brain PI(4)P, 98% DOPC)	This paper	
$PI(3,5)P_2$ transfer<br>L_D: (4% $PI(3,5)P_2$, 2% Rhod-PE, 94% DOPC)	This paper	
$PI(3,5)P_2$ transfer<br>L_A: (100% DOPC)	This paper	
$PI(3,5)P_2$ transfer – equilibrium condition<br>L_D: (2% $PI(3,5)P_2$, 2% Rhod-PE, 96% DOPC)	This paper	
$PI(3,5)P_2$ transfer – equilibrium condition<br>L_A: (2% $PI(3,5)P_2$, 98% DOPC)	This paper	
$PI(3,4,5)P_3$ transfer<br>L_D: (4% $PI(3,4,5)P_3$, 2% Rhod-PE, 94% DOPC)	This paper	
$PI(3,4,5)P_3$ transfer<br>L_A: (100% DOPC)	This paper	
$PI(3,4,5)P_3$ transfer – equilibrium condition<br>L_D: (2% $PI(3,4,5)P_3$, 2% Rhod-PE, 96% DOPC)	This paper	
$PI(3,4,5)P_3$ transfer – equilibrium condition<br>L_A: (2% $PI(3,4,5)P_3$, 98% DOPC)	This paper	
NBD-PE transfer<br>L_D: (4% NBD-PE, 2% Rhod-PE, 94% DOPC)	This paper	
NBD-PE transfer<br>L_A: (100% DOPC)	This paper	

NBD-PA transfer<br>L_D: (4% NBD-PA, 2% Rhod-PE, 94% DOPC)	This paper	
NBD-PA transfer<br>L_A: (100% DOPC)	This paper	
NBD-ceramide transfer<br>L_D: (4% NBD-ceramide, 2% Rhod-PE, 94% DOPC)	This paper	
NBD-ceramide transfer<br>L_A: (100% DOPC)	This paper	
PS transfer<br>L_D: (4% brain PS, 2% Rhod-PE, 94% DOPC)	This paper	
PS transfer<br>L_A: (100% DOPC)	This paper	
PS transfer – equilibrium condition<br>L_D: (2% brain PS, 2% Rhod-PE, 96% DOPC)	This paper	
PS transfer – equilibrium condition<br>L_A: (2% brain PS, 98% DOPC)	This paper	
DHE transfer<br>L_D: (10% DHE, 90% DOPC)	This paper	
DHE transfer<br>L_A: (2.5% DNS-PE, 97.5% DOPC)	This paper	
