# Supplementary material for: PDZD-8 and TEX-2 regulate endosomal PI(4,5)P2 homeostasis via lipid transport to promote embryogenesis in C. elegans
Source: Nat Commun. 2021 Oct 18;12:6065. doi: 10.1038/s41467-021-26177-z (PMC8523718; doi:10.1038/s41467-021-26177-z)
Supplement: Supplementary file 14 — Description of additional supplementary files [file 41467_2021_26177_MOESM14_ESM.docx]

Description of additional supplementary files

Title: Supplementary Movie 1.

Description: Equatorial planes of representative early embryos from (left) wild-type control (WT) and (right) mutant lacking all the four SMP proteins [QKO *(pdzd-8; tex-2; tmem-24; esyt-2)*], expressing PI(4,5)P_2_ biosensor (mCherry::PH^PLCδ1^). Images were taken every 5 s using spinning disc confocal (SDC) microscopy. Image size, 56.54 µm x 39.82 µm.

Title: Supplementary Movie 2.

Description: Equatorial planes of a representative early embryo from a mutant lacking all the four SMP proteins [QKO *(pdzd-8; tex2; tmem-24; esyt-2)*], co-expressing PI(4,5)P_2_ biosensor (mCherry::PH^PLCδ1^) and NMY-2-tagged with GFP (NMY-2::GFP). Images were taken every 5 s using SDC microscopy. Image size, 56.54 µm x 39.82 µm.

Title: Supplementary Movie 3.

Description: Equatorial planes of representative early embryos from (left) wild-type control (WT), (middle) mutant lacking all the four SMP proteins [QKO *(pdzd-8; tex-2; tmem-24; esyt-2)*] and (right) mutant lacking PDZD-8 and TEX-2 [DKO *(pdzd-8; tex-2)*], expressing NMY-2-tagged with GFP (NMY-2::GFP). Images were taken every 5 s using SDC microscopy. Image size, 56.54 µm x 39.82 µm.

Title: Supplementary Movie 4.

Description: Equatorial planes of representative early embryos from (right) OCRL-1 RNAi-treated *unc-26* mutants and (left) OCRL-1 RNAi treated *unc-26;* DKO *(pdzd-8; tex-2)* mutants, co-expressing PI(4,5)P_2_ biosensor (mCherry::PH^PLCδ1^) and NMY-2-tagged with GFP (NMY-2::GFP). Images were taken every 5 s using SDC microscopy. Image size, 56.54 µm x 39.82 µm.

Title: Supplementary Movie 5.

Description: A representative PI(4,5)P_2_-positive vesicle from an early embryo from OCRL-1 RNAi-treated *unc-26;* DKO *(pdzd-8; tex-2)* mutants, co-expressing mCherry::PH^PLCδ1^ (red) and NMY-2::GFP (green). Images were taken every 5 s using SDC microscopy. Image size, 6.05 µm x 5.324 µm.

Title: Supplementary Movie 6.

Description: A representative late endosome from wild-type animals, expressing PDZD-8::mNeonGreen (green) and mCherry::RAB-7 (red) both from endogenous loci. Images were taken every 5 s using SDC microscopy. Image size, 5.28 µm x 4.18 µm.

Title: Supplementary Movie 7.

Description: Equatorial planes of representative early embryos from indicated mutants, expressing NMY-2-tagged with GFP (NMY-2::GFP). Images were taken every 5 s using SDC microscopy. Image size, 55.55 µm x 40.92 µm.

Title: Supplementary Movie 8.

Description: A representative PI(4,5)P_2_-positive vesicle from OCRL-1 RNAi-treated *unc-26;* QKO mutants, expressing mCherry::PH^PLCδ1^ (red) and GFP::RAB-7 (green). Images were taken every 5 s using SDC microscopy. Image size, 4.18 µm x 4.51 µm.

Title: Supplementary Movie 9.

Description: Representative PI(4,5)P_2_-positive vesicles from OCRL-1 RNAi-treated *unc-26;* QKO mutants, expressing mCherry::PH^PLCδ1^ (red) and GFP::RAB-5 (green). Images were taken every 5 s using SDC microscopy. Image size, 4.18 µm x 4.51 µm.

Title: Supplementary Movie 10.

Description: A representative PI(4,5)P_2_-positive vesicle from OCRL-1 RNAi-treated *unc-26;* QKO mutants, expressing mCherry::PH^PLCδ1^ (red) and GFP::RAB-11.1 (green). Images were taken every 5 s using SDC microscopy. Image size, 4.18 µm x 4.51 µm.

Title: Supplementary Movie 11.

Description: Equatorial planes of representative early embryos from (left) wild-type control (WT) and (right) OCRL-1 RNAi-treated *unc-26;* DKO *(pdzd-8; tex-2)* mutants, expressing CAV-1 tagged with GFP (CAV-1::GFP). Images were taken every 5 s using SDC microscopy. Image size, 56.10 µm x 38.39 µm.
